# Xeroderma Pigmentosum Complementation Group C (XPC): Emerging Roles in Non-Dermatologic Malignancies 

**DOI:** 10.3389/fonc.2022.846965

**Published:** 2022-04-21

**Authors:** Nawar Al Nasrallah, Benjamin M. Wiese, Catherine R. Sears

**Affiliations:** ^1^ Division of Pulmonary, Critical Care, Sleep and Occupational Medicine, Department of Medicine, Indiana University School of Medicine, Indianapolis, IN, United States; ^2^ Division of Pulmonary Medicine, Richard L. Roudebush Veterans Affairs Medical Center, Indianapolis, IN, United States

**Keywords:** nucleotide excision repair (NER), base excision repair (BER), lung cancer, biomarker, bladder cancer, chemotherapy, xeroderma pigmentosum (XP)

## Abstract

Xeroderma pigmentosum complementation group C (XPC) is a DNA damage recognition protein essential for initiation of global-genomic nucleotide excision repair (GG-NER). Humans carrying germline mutations in the *XPC* gene exhibit strong susceptibility to skin cancer due to defective removal *via* GG-NER of genotoxic, solar UV-induced dipyrimidine photoproducts. However, XPC is increasingly recognized as important for protection against non-dermatologic cancers, not only through its role in GG-NER, but also by participating in other DNA repair pathways, in the DNA damage response and in transcriptional regulation. Additionally, XPC expression levels and polymorphisms likely impact development and may serve as predictive and therapeutic biomarkers in a number of these non-dermatologic cancers. Here we review the existing literature, focusing on the role of XPC in non-dermatologic cancer development, progression, and treatment response, and highlight possible future applications of XPC as a prognostic and therapeutic biomarker.

## Introduction

Genomic instability from altered DNA repair processes is a hallmark of cancer, playing an important role in both tumor development and treatment response (1). Importantly, the therapeutic efficacy of many chemotherapy drugs and radiation relies on the induction of DNA damage as a means of selectively eliminating rapidly proliferating tumor cells. (2).

Daily DNA damage comes from a variety of different sources exogenous to the cell, such as ultraviolet (UV) light, tobacco smoking, and other chemicals, as well as endogenous sources such as oxidative stress caused by normal cellular metabolism (3). The nucleotide excision repair (NER) pathway is the primary DNA repair pathway involved in repair of bulky, helix distorting intrastrand DNA crosslinks caused by UV or platinum chemotherapeutics, as well as bulky monoadducts induced by environmental carcinogens including B[*a*]P-7,8-dihydrodiol-9,10-epoxide (BPDE) and aflatoxin B1 (AFB1). Much of our understanding of NER comes from studying the repair of UV-induced lesions, such as pyrimidine-pyrimidone (6-4) photoproduct (6–4PPs) and cyclobutane pyrimidine dimers (CPDs), for which NER serves as the primary repair pathway (4). Critical to its role in cancer therapeutic response, NER is the primary repair pathway for 1,2-d(GpG) and 1,3-d(GpTpG) intrastrand platinum crosslinking lesions, the predominant DNA adducts produced by the commonly used chemotherapeutic drugs cisplatin and carboplatin (5). The NER pathway consists of 4 essential steps: recognition, incision/excision, re-synthesis, and ligation (2). Differing in the mechanism of DNA damage recognition, NER is divided into two subpathways: global genomic NER (GG-NER) and transcription-coupled NER (TC-NER). Both NER subpathways repair helix-destabilizing DNA lesions, with TC-NER rapidly repairing damage in actively transcribed genes. TC-NER is initiated when the RNA polymerase II complex is physically stalled at the site of a DNA damaging lesion; this subsequently triggers recruitment of CSB and coordinated recruitment of other TC-NER recognition proteins including CSA, XAB2, UVSSA, USP7 and others (6, 7). Initiated by the xeroderma pigmentosum group C (XPC) complex, GG-NER recognizes helix-distorting lesions anywhere throughout the genome but is primarily responsible for the slower repair of damage on non-transcribed portions (8, 9). Following damage recognition, subsequent NER repair then progresses identically between both NER subpathways. XPC is critical to damage recognition and initiation of GG-NER, but dispensable for TC-NER (9).

There is a clear and established association between defective NER and tumor development, as illustrated by the rare autosomal recessive congenital syndrome xeroderma pigmentosum (XP). XP patients are characterized by defective nucleotide excision repair (NER) of sunlight-induced dipyrimidine photoproducts (10). Depending on the mutated NER protein, XP patients present with a spectrum of disease, which consists of various neurological degenerative disorders and even developmental defects, but all XP patients present with extreme photosensitivity and a strong predisposition to skin cancer (10, 11). Those with a mutation in XPC (XP-C), a common cause of XP in Europe, the United States and North Africa, present with classical XP skin manifestations, including photosensitivity and early dermatologic malignancies, without neurological or developmental defects (11, 12). Indeed, both non-melanomatous skin cancers and melanomas develop more often (10,000 and 2,000-fold increased incidence) and at a much younger age in XP compared to non-XP populations, with a median age at diagnosis of 9 and 22 years respectively (13). Importantly, although XP patients most commonly die of skin cancers or of progressive neurologic diseases, internal malignancies are frequently described in XP patients, with a 39-year prospective cohort study finding internal cancers as the cause of death in 17%, highlighting an important role of NER in non-dermatologic malignancies as well (13, 14).

XPC is increasingly recognized as an important player in solid organ cancer development and response to cancer therapeutics, both through its canonical role in GG-NER and through other repair pathways. Here we review the most recent updates on the role of XPC in non-dermatologic malignancies.

## XPC Role in DNA Repair

### XPC in GG-NER

XPC is essential to GG-NER, serving as the primary initiating factor. XPC scans the genome in a 5’-to-3’ directionality until it detects strand distortion caused by DNA damaging lesions, binding the opposite strand in a sequence-independent manner (8, 15, 16). The XPC protein *in vivo* is found in a heterodimeric form with RAD23B (human orthologue HR23B) which further stimulates XPC’s role in NER repair (17). Centrin2 forms a heterotrimer with XPC/HR23B, which has been found to augment binding to DNA damage sites (18). While the XPC complex is typically sufficient to identify NER-repaired DNA lesions, some minimally strand-distorting lesions, such as UV-induced CPD, require recognition by DDB2 and DDB1, which then recruit XPC to the damage site (7).

After the initial recognition of a helix distorting lesion by either XPC or RNA polymerase II, NER proceeds in a stepwise sequence that involves recruitment of several proteins. Transcription factor IIH complex (TFIIH) partially unwinds the DNA duplex at the site of DNA damage, creating an opened bubble (16, 19). TFIIH further coordinates repair by interacting with XPA, stabilizing the bubble along with the single stranded binding protein RPA, and finally engaging with the nuclease (XPF/ERCC1) that makes an incision 5’ of the lesion. Subsequent repair involves coordination of repair synthesis by DNA polymerases δ, ϵ or κ, subsequent incision 3’ of the DNA lesion by XPG to remove the damaged strand, and finally repair of the nick by DNA ligases. Several excellent reviews are available which expand upon and provide excellent graphical representation of the steps involved in NER (7, 16, 20).

A number of recent studies highlight that post-translational modifications of XPC, including polyubiquitination, SUMOylation and phosphorylation, likely impact XPC efficiency to detect DNA damage and initiate NER (21–25). Polyubiquitination of XPC appears to aid in repair of UV-damaged DNA, by allowing XPC to replace DDB1/DDB2 proteins and in promoting XPC binding to the site of DNA damage (21, 25). Tight control of XPC ubiquitination is likely required to ensure DNA repair and may be dysregulated in human cancers. For instance, overexpression of ubiquitin ligases, such as Cullin-RING ubiquitin ligase 4 A (CUL4A), is common in cigarette smoke-related lung cancer, and inversely correlates to XPC expression (26). SUMOylation of XPC appears to stabilize the protein, preventing proteasome degradation and enhancing GG-NER in the setting of UV-induced DNA damage (22). XPC phosphorylation is closely regulated after DNA damage, with phosphorylation at serine 982 likely mediated by the DNA damage response proteins ATM and ATR, and dephosphorylation mediated by wild-type p53-induced phosphatase 1 (WIP1) (27, 28). Following UVB exposure, serine/threonine casein kinase 2 (CK2) phosphorylates XPC at serine 94, leading to recruitment of ubiquitinated XPC and downstream NER factors to DNA damage sites (24). Phosphorylation of XPC at serine 892 seems to decrease repair of UVB-induced DNA damage, including CPD and 6-4PP, while serine 94 phosphorylation promotes GG-NER repair (24). However, whether these modifications impact the role of XPC on other repair pathways, or how they affect XPC’s role in repair of DNA damage from other sources, such as cisplatin, is not well-studied. Further, modification of other proteins may impact XPC function. For instance, histone acetylation may decrease NER through attenuated XPC interaction at sites of DNA damage (23). These modifications, which regulate XPC function in GG-NER repair and the downstream DNA damage response, are likely to impact cancer risk and response to therapy, although this specific link requires more study.

### XPC in Other DNA Repair Processes

**Figure 1:**
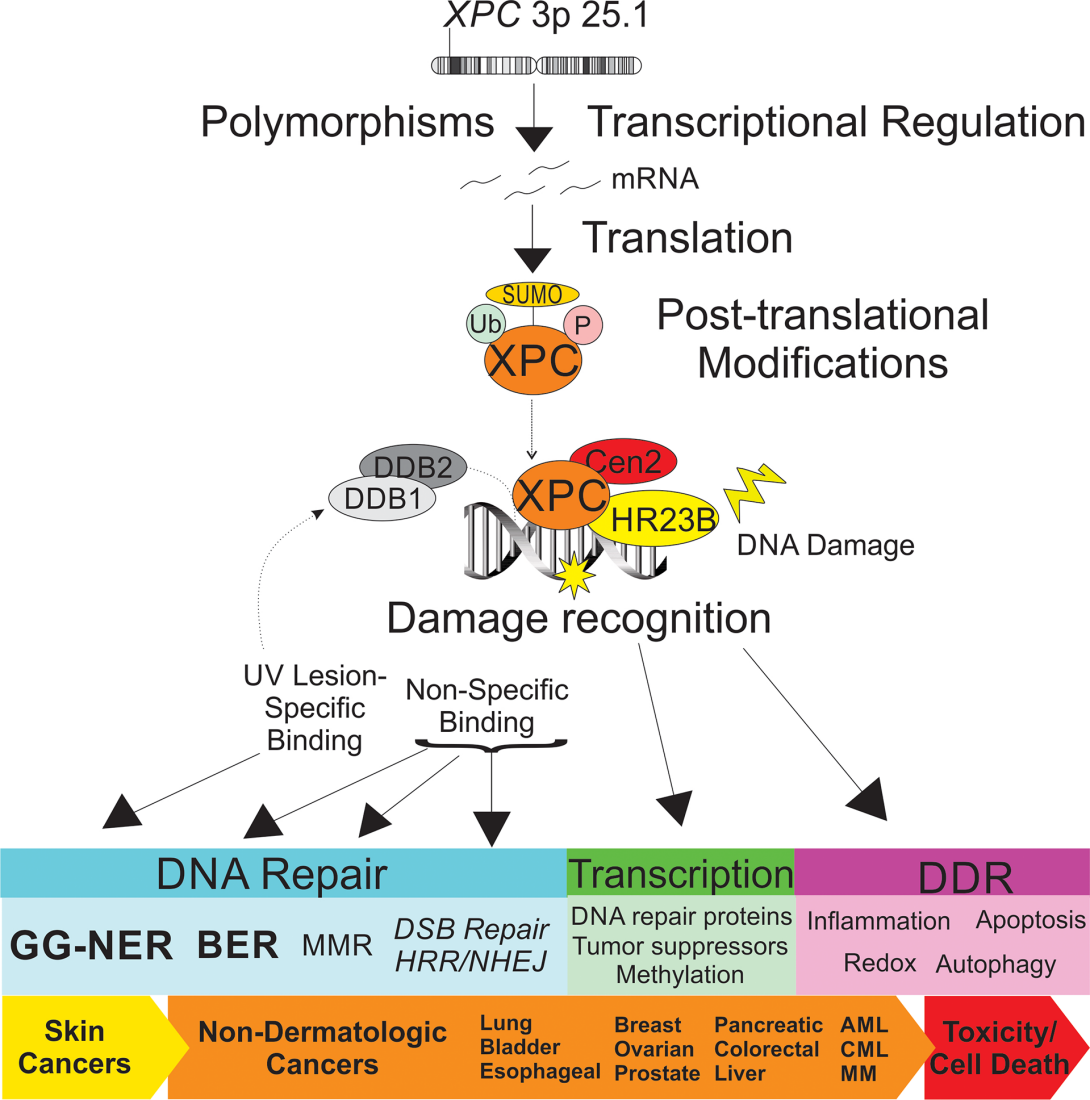
Schematic representation of the impact of XPC in dermatologic and non-dermatologic malignancies. Both *XPC* mutations and transcriptional regulation of *XPC* expression levels are described as impacting risk of the cancer development and response to treatment. Post-translational modifications of XPC include ubiquitination, SUMOylation and phosphorylation, which impact XPC expression levels and XPC function. XPC is a versatile DNA damage sensor, leading to differing binding affinities and DNA-XPC conformational changes for UV-induced DNA damage (“specific binding”, in concert with the UV-DDB complex, leading to GG-NER) and other DNA damage (“non-specific binding”, leading to other DNA repair pathways). Differential response of XPC to DNA damage leads to classical GG-NER or alternate DNA repair, altered transcriptional regulation, and DNA damage response ultimately impacting cancer risk and tumor cell toxicity. XPC, xeroderma pigmentosum group c; Ub, ubiquitin; SUMO, small ubiquitin-like modifier; P, phosphorylation site; DDB1, DNA damage-binding 1; DDB2, DNA damage binding 2; Cen2, centrin 2; HR23B, human UV excision repair protein RAD23; GG-NER, global genomic nucleotide excision repair; BER, base excision repair; MMR, mismatch repair; DSB, double strand break; HRR, homologous recombination repair; DDR, DNA damage response; AML, acute myeloid leukemia; CML, chronic myeloid leukemia; MM, multiple myeloma.

It is important to note the mounting evidence highlighting an important link between the role of XPC in DNA repair, DNA damage response and transcriptional regulation and cancer development. These are summarized in **Figure 1**. In particular, the impact of XPC DNA damage repair extends beyond its canonical role in GG-NER. XPC may play a role as a more global DNA damage sensor. Recent *in vitro* studies have elucidated a role of Rad4, the yeast homolog of XPC, in the recognition and repair of multiple contiguous mismatched base pairs (29). Specifically, *in vitro* binding and conformational studies suggest that Rad4/XPC interacts with the nucleotides directly across from the mismatched bases (on the complementary strand), leading to subsequent unwinding, DNA bending, and flipping out of the mismatched nucleotides and stabilization of this conformation to allow for subsequent DNA repair (29, 30). These studies suggest a mechanism by which XPC acts as a universal DNA damage sensor, recognizing sites of DNA distortion and binding in a lesion-agnostic fashion (“non-specific binding”). Indeed, recent studies suggest that the Rad4/XPC-DNA binding leads to different conformational changes based on the lesion type, such that XPC bound at the site of UV-induced DNA damage (“specific binding”) facilitates recruitment and initiation of NER while “non-specific” binding to minimally strand-distorting lesions facilitates non-NER repair (29, 30). Extensive structural analysis has been done to understand sequence and structural changes of DNA lesions sensitive and resistant to Rad4/XPC binding and subsequent GG-NER efficiency (31).

Mounting evidence points to a role of XPC in base excision repair (BER). BER is the primary repair mechanism of small, base modifications that do not distort the DNA helical structure. Fibroblasts obtained from XP-C patients displayed increased oxidative DNA damage after UVB-irradiation compared to fibroblasts without an *XPC* defect. These UV-treated *XPC* deficient fibroblasts had decreased gene expression of a number of factors involved in BER, including *OGG1*, *MYH*, *APE1*, *LIG3*, *XRCC1*, and *Polβ*, and this correlated with decreased protein expression in three BER-glycosylases: OGG1, MYH, and APE1 (32). Likewise, *XPC* deficient fibroblast cell lines show lower levels of *APE1* and *OGG1* mRNA compared to *XPC* proficient cells, however transiently complementing these cells with XPC only augmented the level and function of OGG1 but not APE1, suggesting a differential impact of XPC on OGG1 glycosylase activity (33). Numerous *in vitro* studies support a role of XPC in augmenting BER activity, particularly through augmentation of the glycosylase activities of OGG1, SMUG1, 3-methyladenine DNA glycosylase (MPG) and thymine DNA glycosylase (TDG) (34–37). XPC may also augment BER through DNA damage recognition. Interestingly, live cell imaging studies show a rapid recruitment of both cockayne syndrome protein B (CSB, involved in TC-NER) and XPC to the BER-repaired 8-dihydro-8-oxodeoxyguanosine (8-OHdG) DNA lesion, suggesting a role of XPC in early recognition of BER-repaired lesions, even though these do not cause significant strand distortion (38). This may be further explained by the recent finding that DDB2 rapidly localizes to 8-OHdG lesions, preceding and augmenting XPC and subsequent OGG1 recruitment (39). This role of DDB2 in recruiting XPC to minimally helix-distorting lesions is similar to that modeled in GG-NER repair. Interestingly, this recent study suggested a specific role of XPC and DDB2 in augmenting OGG1-mediated BER repair of 8-OHdG lesions in non-transcribed, heavily chromatin-bound genomic regions, which differed from the mechanism observed for repair of 8-OHdG lesions in actively transcribed regions, which ultimately involved recruitment of XPA by OGG1 but was independent of XPC and DDB2 (39). *In vivo* studies further support a supportive role of XPC in BER. *Xpc* deficient mice had increased oxidative stress and mutation load over time with treatment with pro-oxidant agents, which was not observed in *Xpa* deficient and wild type mice (40). However, there was a comparable increase of 8-OHdG lesions by liquid chromatography electrospray tandem mass spectrometry in the uterus of both *Xpc* deficient and *Xpc* proficient mice after treatment with equine estrogen, suggesting the effect may be specific to the damaging agent, duration of treatment or tissue-specific (41). Urethane-treated *Xpc*-/- mice developed an increase in lung adenocarcinomas compared to their wild-type counterparts, but treatment with the anti-oxidant N-acetylcysteine (NAC) decreased tumor development, further supporting a link between XPC, oxidative damage and cancer development (42). Although modified base recognition and augmentation of BER glycosylase and APE1 endonuclease activity have all been proposed, exactly how XPC is involved in BER of oxidized DNA lesions and the subsequent cancer development remain areas of active research.

Mismatched DNA nucleotides, particularly those occurring during replication, are repaired by DNA mismatch repair (MMR). In humans, deficient MMR, through both sporadic and inherited genetic disease, is linked to aging and cancer by promoting genomic instability (43, 44). In particular, defective MMR leads to Lynch syndrome, characterized by a high lifetime risk of colon and other cancer, and MMR defects are associated with ~10-20% of sporadic colon cancers (45, 46). Increasingly, cooperative and possibly overlapping roles of both MMR and NER proteins have been implicated in the recognition and repair of some DNA interstrand crosslinks (ICLs), one of the most cytotoxic types of DNA damage. ICLs are caused by a number of environmental toxins as well as commonly used chemotherapeutic agents, including cisplatin, carboplatin and oxaliplatin commonly used to treat solid-organ tumors (47). Repair of these lesions requires cooperation between different DNA repair pathways, including the Fanconi anemia (FA), NER, homologous recombination repair (HRR) and translesion synthesis (TLS) pathways (47). XPC, along with other NER proteins, were found to be essential for repair of site-specific ICLs caused by psoralen and mitomycin C *in vitro* using a host-cell reactivation assay (48). Further, both the MMR and NER pathways have been implicated in the repair of triplex-forming oligonucleotide (TFO)-directed psoralen ICLs (Tdp-ICLs) (49–52). Specifically, in MSH2-deficient human cell-free extracts, both binding by the XPC complex and repair of Tdp-ICLs were decreased, further highlighting a cooperative role between NER and MMR ICL repair (53, 54). Additionally, two NER protein complexes, XPC-Rad23B and XPA-RPA can bind psoralen ICLs in cells and *in vitro*, forming a complex with the MMR complex MutSβ, without which cell toxicity to psoralen increases (55). Further evidence of a connection between XPC and MMR is evidenced in cisplatin-treated *XPC* deficient cells, in which altered expression was noted in three MMR genes: *MLH1*, *MSH2*, and *MSH6* (56). Cells deficient in *Xpa* and *Msh2* are less sensitive to UV-induced cellular toxicity compared to *Xpa*-/- cells with normal *Msh2* expression, suggesting a role of MSH2 in the DNA damage response but not necessarily in NER repair of UV-induced DNA damage (57). Finally, combined defects in NER and MMR have been associated with increased UV-induced skin cancers. Combined *Xpa* and *Msh2* deficiencies in mice are associated with an increase in UV-induced skin cancers, and similarly *Xpc*-/-; *Msh2-/-* mice developed UV-induced skin cancers earlier than their wild-type counterparts or those deficient in either *Xpc* or *Msh2* alone, suggesting cooperative but non-overlapping roles in UV-induced DNA damage repair (57, 58). An XPC-deficient lymphoblastoid cell line modified by acquired tolerance to the MMR-dependent chemical N-methyl-N-nitrosourea (MNU) exhibited decreased MSH6 expression and MMR efficiency (59). These XPC-deficient, MSH6-low cells effectively repaired UV- and cisplatin-induced lesions by TC-NER, suggesting that the previously observed MMR-NER interactions may rest in interactions with proteins involved in GG-NER, particularly in cancer development. Of interest, the authors of this study noted unusual difficulty in producing MMR deficient variants in two XPC-deficient lymphoblastoid cell lines, further suggesting possible yet still undefined interactions between XPC and MMR functions. Overall, these findings suggest that XPC may cooperate with MMR proteins in the identification and repair of strand-distorting configurations of mismatched nucleotides and ICLs and may serve a role in regulation of the MMR pathway for some types of DNA damage, impacting of mutagenesis.

Additionally, XPC may play a role in DNA double strand break (DSB) repair. Long-term *XPC* knock-down in HeLa cells was associated with increased sensitivity to the chemotherapeutic drug, etoposide, the cytotoxicity of which is dependent on replication-induced DSB; gamma-irradiation of these cells lead to cell cycle alterations without altered clonogenic survival (60). Furthermore, the increased somatic and germ line mutation rates, as measured by expanded simple tandem repeat (ESTR), were increased in *Xpc* deficient mice exposed to whole body irradiation (61). More direct evaluation of NHEJ activity *in vitro* using Manley extracts from *XPC* knock-down HeLa cells showed a capacity of NHEJ rejoining with linear but not circular DNA (60). XPC deficiency has also been associated with inhibition of BRCA1 expression on bladder cancer cells treated with cisplatin, resulting in accumulation of DNA damage and pointing to a potential indirect role of XPC in homologous recombination or, more likely, replication-induced double strand breaks (62). Overall, this suggests a complex, likely indirect role of XPC in the repair of multiple types of DNA damage.

The impact of XPC in DNA damage is not solely associated with its roles in DNA repair but has been implicated in altered downstream DNA damage response (**Figure 1**). For instance, at sites of UV-induced DNA damage, XPC attracts and physically interacts with Ataxia telangiectasia- and Rad3- related (ATR) and Ataxia telangiectasia mutated (ATM) proteins, two kinases important in DNA damage- and replication stress-induced checkpoint activations. Both DDB2 and XPC facilitate ATR and ATM phosphorylation and subsequent activation, leading to phosphoactivation of ATR- and ATM- substrates involved in cell cycle regulation (including Chk1 and Chk2) (63). Additionally, XPC facilitates ATR- and ATM- recruitment to sites of DNA damage as well as two proteins, BRCA1 and RAD51, known to be involved in replication and HRR (63). XPC has been implicated in enhancing DNA damage–induced apoptosis through inhibition of caspase-2 transcription (64), and both increased apoptosis and altered autophagy are observed in cells exposed to carcinogenic cigarette smoke and arsenic trioxide *in vitro* and *in vivo* (65, 66). Independent of DNA damage, *XPC* silencing and overexpression in mouse and human embryonic stem cell models support a role of XPC in global DNA demethylation through augmentation of TDG avidity (37, 67). XPC may have an even broader role on transcriptional regulation through coordination with other transcription factors and has been linked with regulation of a number of genes, including tumor suppressor genes, even in the absence of DNA damage (37, 68, 69). XPC involvement in the DNA damage response may also impact cell redox homeostasis and also in local inflammation. For instance, silencing of XPC in arsenic trioxide-treated human glioma cells was associated with decreased anti-oxidant factors and subsequent increase in oxidative damage, including 8-OHdG (65). Melis and colleagues described the glutathione anti-oxidant response as deficient in *Xpc*-/- mice, and most recently, Mori and colleagues describe a redox imbalance due to compromised mitochondrial function and reduced glutathione peroxidase activity (70, 71). Lung fibroblasts exposed to both the carcinogen BPDE and to the chemotherapeutic drug cisplatin produced higher levels of the pro-inflammatory, tumor promoting cytokine interleukin-6 (IL-6) through the p38-SAPK pathway (72). As the local tumor immune response is increasingly recognized as critical to solid organ cancer development, the role of XPC in local tumor microenvironment, including immune escape, warrants further investigation.

## XPC in Hematologic Cancers

The role of XPC in hematopoietic malignancy has been explored over the last several years, both in mouse models and observations in various patient populations. XPC deficient mice (*Xpc-/-*) have a significantly higher frequency of spontaneous mutations in the *hprt* gene in splenic T lymphocytes as compared to *Xpa-/-* and *Csb-/-* mice; this was also enhanced with aging (73). Similarly, long-term exposure to paraquat in *Xpc-/-* mice leads to an increase in lymphoid hyperplasia (40). XPC deficient mice had hypocellular bone marrow associated with a 10-fold increased sensitivity to carboplatin and decreased cell and overall mouse survival as compared to wild type mice, suggesting an important role of XPC in hematopoietic cell response to treatment with platinum-containing drugs (74). Importantly, these studies suggest that XPC expression may impact bone marrow suppression and altered hematopoiesis, common treatment-limiting adverse events associated with platinum-based chemotherapeutic agents.

**Figure 2:**
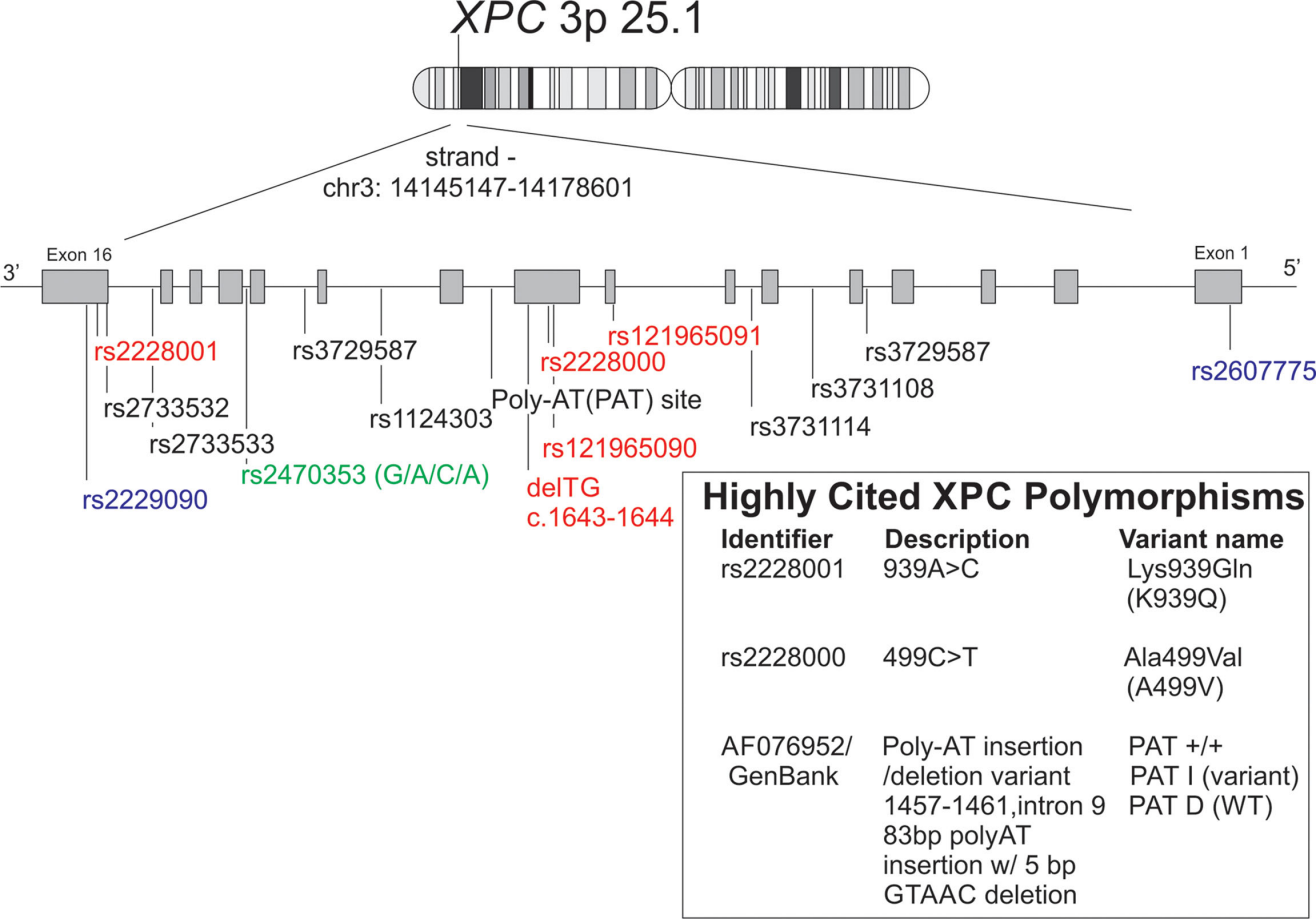
Schematic representation of *XPC* polymorphisms discussed in this manuscript along with alternate names/identifiers for the *XPC* polymorphisms most commonly studied in non-dermatologic cancers. Reference XPC gene (chr 3:p25.1) with polymorphisms was reproduced using the GRCh38 (hg38) sequencing using the UCSC genome browser tool. [(112) http://genome.ucsc.edu. *Accessed 1/30/22*]. Red = missense mutations, blue = 5’ or 3’ UTR variants, green = upstream of transcript variant.

Alterations in DNA repair processes, including those associated with *XPC* deficiency, have been linked to hematologic malignancies in a human population (75). While overshadowed by the recognition of skin malignancies early after identification of the XP phenotype, early case reports include pediatric and young adult XP-C patients who develop hematologic malignancies (14). More recent studies have shown an increased propensity for hematologic malignancies and sarcomas in populations of individuals with xeroderma pigmentosum deficient in XPC (XP-C). Individuals with XP-C are at an increased risk of leukemia and other hematologic malignancies, as well as alterations in genotoxic effects due to treatment of these cancers (76, 77). Sarasin et al. examined a cohort of 161 patients with XP-C and found that 13 of these individuals developed either overt myelodysplastic syndrome (MDS) or acute myeloid leukemia (AML) with a median age of 22 years at diagnosis (**Table 1**). This finding of MDS/AML was specific for the most common homozygous frameshift *XPC* mutation delTG (c.1643_1644delTG; p.Val548Ala>fsX25) and has not been observed with an increased frequency in other XP patients (77). Similarly, a cohort of 117 individuals with XP-C were followed from 1971 to 2018 and four patients were found to develop hematologic malignancies, including MDS, acute leukemias and high grade lymphoma (110). More recently, a shared mutational profile was identified by whole genome sequencing in leukemias from six XP-C patients, which differed from the mutational patterns in non-XP-C spontaneous AML samples and corresponded to a pattern described with altered GG-NER (111). Single nucleotide polymorphisms (SNPs) of the *XPC* gene have been studied in a number of malignancies, many of which may modify disease risk, prognosis or alter treatment response (**Figure 2**). Of these, several have been studied in leukemias (**Table 1**). In AML treated with induction chemotherapy, the *XPC* Ala499Val SNP was associated with lower overall disease-free survival, particularly when combined with an *XPD* codon 751 AC/CC polymorphism (78), and two *XPC* SNPs (Ala499Val and Lys939Gln) were associated with variable responses to imatinib in BCR-ABL driven chronic myelogenous leukemia (CML) (79). In regard to tolerating induction chemotherapy or hematopoietic stem cell transplantation in the setting of XPC abnormalities, there is little data.

**Table 1:** Summary of clinical studies evaluating *XPC* polymorphisms and epigenetic alterations by malignancy.

Malignancy	*XPC* mutation or SNP	Clinical association	Study name and size
AML AML/MDS	*XPC* polymorphism Ala499Val (rs2228000)	*XPC* Ala499Val was associated with lower overall disease-free survival in AML patient treated with induction chemotherapy	(78) 170 adult de-novo AML patients with intermediate cytogenetics treated with induction chemotherapy
	c.1643-1644 delTG *XPC* mutation	Increased risk for developing MDS or AML	(77) 161 patients with XP-C from 142 consanguineous North African families living in France
CML	*XPC* polymorphisms 499C and 939A	Both 499C and 939A wild-type haplotype associated with improved response to imatinib.	(79) 92 Caucasian patients with BCR-ABL-positive CML in five Spanish Institutions.
Multiple Myeloma	*XPC* polymorphism 939A>C (Lys939Gln) (rs2228001)	*XPC* Lys939Gln was associated with freedom from progression (FFP) in patients receiving high-dose melphalan (HDM)	(80) 169 MM patients from France and Canada who underwent treatment with HDM and stem cell transplant.
Lung cancer	*XPC* polymorphism PAT+/+ variant	*XPC* PAT +/+ was associated with an increased risk for lung cancer	(81) Hospital-based case-control study of 359 newly diagnosed lung cancer and matched 375 control subjects in Northern Spain.
	*XPC* Lys939Gln polymorphisms (rs2228001)	Heterozygous carriers of the C-allele and homozygous carriers had higher risk of lung cancer in the youngest available age interval (50–55 years)	(82) Danish study included 265 lung cancer cases and 272 control individuals.
	*XPC* Polymorphisms Lys939Gln and Ala499Val (rs2228001, rs2228000)	*XPC* 939Gln/Gln and 939Lys/Gln both were associated with increased risk of lung cancer with low penetrance. *XPC* 499Val increased total cancer risk (OR1.15), but not specifically the lung cancer.	(83) Meta-analysis that included 33 published case–control studies
	*XPC* Polymorphism Lys939Gln (rs2228001)	Females carrying *XPC* 939Gln/Gln vs. *XPC* 939Lys/Gln. 939Gln/Gln had significantly increased risk of lung cancer as well as other females and males with several combination of polymorphisms in *XPC*, *XPD* (Lys751Gln), *hOGG1* (Ser326Cys) and *XRCC1* (Arg399Gln)	(84) Case-Control study of 382 patients with lung cancer and 379 healthy controls of Caucasian Slovaks race/ethnicity.
	XPC polymorphisms (Lys939Gln, Ala499Val, and PAT) (rs2228001, rs2228000)	Homozygous Gln939Gln genotype was associated with significantly increased risk of lung cancer in Asian population PAT -/- genotype significantly reduced susceptibility to lung cancer in Caucasian population *XPC* Ala499Val polymorphism was not associated with lung cancer risk.	(85) Meta-analysis of 14 studies including 5647 lung cancer cases and 6908 controls
	*XPC* Lys939Gln polymorphism (rs2228001)	*XPC* Lys939Gln was associated with higher lung cancer susceptibility (OR 1.28)	(86) Polymorphism stratified meta-analysis, 16 studies of cancers with 5581 cases and 6351 controls (5 studies specific for lung cancer)
	*XPC* polymorphism rs2733533	XPC rs2733533 associated with lung cancer susceptibility, the combination of genotype A carriers and heavy smokers (≥30 pack-year) had a 13.32-fold risk of lung cancer compared with the C/C genotype and no smoking.	(87) Case control study of 265 lung cancer patients and 301 healthy controls
	*XPC* polymorphisms Lys939Gln, Ala499Val (rs2228001, rs2228000)	Neither SNP altered response to platinum-based chemotherapy.	(88) Meta-analysis of 1,615 patients from 10 studies for the rs2228001 and 858 samples from six studies for rs2228000.
Prostate Cancer (PC)	*XPC* polymorphisms PAT, Lys939Gln (rs2228001)	PAT (insertion/insertion) genotype increases the risk of developing PC, *XPC* Lys939Gln and *XPC*-PAT variants (Lys/Gln + PAT D/D) were protected against PC development compared to controls.	(89) Study in Tunisian population included 110 PC patients compared to 266 matched control men.
	*XPC* PAT polymorphism	*XPC* PAT+/+ subjects genotype exhibited a significantly increased risk for PC, smokers with PAT+/− or PAT+/+ had a higher risk for PC.	(90) 202 subjects with prostate cancer and 221 healthy controls in a Chinese Han population.
	NER polymorphisms, *XPC* intron 11 C>A (rs3729587)	*XPC* intron11 C/A polymorphism was associated with an increased risk of prostate cancer.	(91) Hospital-based cohort consisted of 152 patients with prostate cancer and 142 male controls.
	*XPC* polymorphism (Lys939Gln, PAT) (rs2228001)	*XPC* PAT deletion/insertion (D/I) and insertion/insertion (I/I) could decrease the risk of PC	(92) Iranian cohort including 154 prostate cancer patients and 205 Benign Prostate Hyperplasia (BPH) controls
Ovarian Cancer	*XPC* polymorphisms Ala299Val and Lys939Gln (s2228000 and rs2228001)	*XPC* Ala299Val was associated with reduced risk of ovarian cancer *XPC* Lys939Gln increased risk of ovarian cancer	(93) Chinese cohort, 89 ovarian cancer patients 356 cancer-free women
	*XPC* polymorphisms rs3731108, rs1124303 and PAT	*XPC* SNP rs3731108 (AG)/AA versus the GG genotype, SNP rs1124303 (GT)/GG genotype versus TT genotype and PAT (-/+)/(-/-) genotype versus the (+/+) genotype were associated with a prolonged PFS	(94) 139 patients with stage III and IV papillary serous ovarian cancer who underwent primary cytoreductive surgery followed by platinum-based chemotherapy.
Bladder Cancer (BC)	*XPC* Ala499Val polymorphism (rs2228000)	Ala499Val showed an increased overall cancer risk (OR 1.15), and specifically for BC in the simple genetic model	(83) meta-analysis that included 33 published case–control studies
	*XPC* polymorphisms (rs2228000)	*XPC* Ala499Val associated with increased BC susceptibility (OR 1.33)	(86) Polymorphism stratified meta-analysis, 11 published case-control studies of cancer with 5581 cases and 6351 controls
	*XPC* Ala499Val polymorphism (rs2228000)	Associated with risk of XPC Ala499Val associated with increased by 3 different calculations (allelic contrast, OR 1.11; homozygote comparison, OR 1.35; recessive genetic model, OR 1.36)	(95) Meta-analysis of 13 case-control studies, 4,927 bladder cancer cases and 5185 controls
	*XPC* polymorphisms Lys939Gln, Ala499Val, PAT (s2228000, rs2228001, PAT)	Multiple models showing increased BC susceptibility with *XPC* Lys939Gln, Ala499Val and PAT-/+ polymorphisms. Suggested polymorphism risk stratification may differ based on Asian vs Caucasian populations.	(96) Meta-analysis, 14 case-control BC studies, 10 Lys939Gln (3,934 cases, 4,269 controls), 5 Ala499Val (2,113 cases, 2,249 controls), 7 PAT-/+ (2,834 cases, 3,048 controls)
	*XPC* polymorphisms Lys939Gln, Ala499Val, PAT (s2228000, rs2228001)	Suggested increased bladder cancer risk with Ala499Val but not Lys939Gln. Lys939Gln bladder cancer risk appeared related to tobacco smoking or chewing (OR 2.23 and 2.4)	(97) Meta-analysis, 18 case-control BC studies, 7 studies Ala499Val (2893 cases, 3056 controls), 11 studies Lys939Gln (5064 cases, 5208 controls)
	Rare *XPC* polymorphisms (rs121965091, rs121965090)	4 of 5 novel *XPC* variants (Phe302Ser, Arg393Trp, c*156G>A, c.2251-37C>A) associated with increased BC odds (OR 3.1 for having 1+ variant)	(98) Case-control, 771 BC cases and 800 controls
	XPC mRNA and protein expression	Low XPC expression associated with increased BC recurrence and decreased survival	(99) mRNA: 79 BC patients, IHC: 219 BC patients. Relapse at 2 years, survival at time of publication (min-3 years, max 12 years)
Pancreatic cancer	*XPC* polymorphism PAT	PAT +/+ genotype could protect against pancreatic carcinogenesis.	(100) Study included 101 incident cases with pancreatic cancer and 337 controls
	*XPC* tagging SNPs rs2470353, rs2607775, rs2228000, rs3731114 and rs3729587.	For rs2470353, pancreatic cancer risk was increased in subjects with GC and GC+CC gene types Compared with the GG gene type. For rs2607775 the CG and CG+GG gene types were associated with increased pancreatic cancer risk compared with the CC gene type. CCC haplotype of rs2228000, rs3731114 and rs3729587 associated with an increased pancreatic cancer risk	(101) Study included 205 pancreatic cancer cases and 230 controls.
Esophageal cancer	Genetic variants of *XPA* in 50UTR and *XPC* at K939Q (rs2228001)	*XPA* 50UTR A/G and *XPC* K939Q C/C genotypes associated with a higher risk of mortality after treatment compared with wild-type homozygous genotypes especially in the population treated with esophagectomy and undergoing concurrent neoadjuvant chemoradiotherapy.	(102) 501 patients with esophageal squamous cell carcinoma (ESCC).
	*XPC* PAT polymorphism	*XPC* PAT -/+ genotype associated with decreased esophageal cancer risk	(103) 387 White esophageal patients and 462 White controls matched
	Multiple SNP panel, included *XPC* polymorphisms 499CC and 939AC+CC	5-polymorphism panel (*MTHFR* 677TT, *MDR1*2677GT, *GSTP1* 114CC, *XPC* 499CC, *XPC* 939AC+CC) that has a 79% sensitivity and 85.4% specificity of predicting 5 years PFS. They were associated to shorter RFS and in a univariate analysis.	(104) 124 patients receiving neoadjuvant chemoradiation treatment for locally advanced esophageal cancer
Colorectal Cancer and Adenomas (CRC)	*XPC* SNPs (various) (rs2228001)	Haplotype *XPC* A499V independently protective from smoking-associated risk of CRC	(105) 772 subjects with left-sided advanced adenoma vs 777 Controls.
	XPC mRNA and protein expression	High XPC expression might be predictive of survival in CRC	(106) 167 patients with colorectal cancer
Breast Cancer	*XPC* polymorphisms K939Q (rs2228001) and rs2733532	rs2228001–A > C and rs2733532–C > T are associated with an increased risk for breast cancer development	(107) 493 breast cancer cases and 387 controls
	*XPC* polymorphisms Lys939Gln and PAT (rs2228001)	PAT -/+ is associated with an increased risk of breast cancer Combined genotypes 939AC/PAT+/+ and 939CC/PAT+/+ are associated with an increased risk of breast cancer.	(108) 200 women diagnosed with breast cancer as cases and 200 ethnically matched healthy controls
Hepatocellular Carcinoma	*XPC* polymorphism Lys939Gln (rs2228001)	Lys939Gln allele differed in HCC risk, with risk of *XPC*-GG > *XPC*-LG > *XPC*-LL. Heterozygous *XPC* 939LG and/or homozygous *XPC* 939GG, compared to homozygous *XPC* 939LL was associated with shorter overall survival	(109) 1156 HCC cases and 1402 controls without liver disease

RFS, relapse free survival; PFS, progression free survival; PC, prostate cancer; BC, bladder cancer.

Recently, the NER pathway has been studied in the setting of multiple myeloma (MM) due to the reliance on alkylating agents in the treatment of this malignancy; DNA damage caused by alkylating agents are typically repaired by NER. Dumontet et al. found that SNPs in multiple genes, including *XPC*, were associated with longer time to progression- in individuals treated with vincristine-adriamycin-dexamethasone followed by high dose melphalan and stem cell transplantation (80). Similarly, inhibition of the NER pathway in multiple myeloma increases the sensitivity to alkylating agents and overcomes resistance to these alkylating agents (113). Though XPC has not been explicitly implicated in these latter studies, it warrants further investigation given the role of the NER pathway and reliance on alkylating agents in multiple myeloma.

## XPC in Solid Cancers

### Lung Cancer

Lung cancer is characterized by some of the highest levels of genomic diversity, and alterations in DNA repair pathways, including NER, have been proposed to play a role in lung cancer development (114, 115). Although dominated by dermatologic malignancies, early series of XP-C patients reveal cases of bronchogenic lung carcinomas (14, 116). Germline mutations causing XP-C are rare, however, more common *XPC* polymorphisms and variations in gene expression have been studied in lung cancer (**Table 1**). In the most common subset of lung cancers, non-small cell lung cancer (NSCLC), decreased tumor *XPC* mRNA level has been associated with poor outcomes (117).

Numerous studies associate various *XPC* SNP polymorphisms with lung cancer development, which, among other factors, may be influenced by gender and cigarette smoking status (**Table 1**) (81–87), and many *XPC* polymorphisms have been found to functionally modulate DNA repair capacity (118). It is likely that epigenetic regulation leads to decreased *XPC* gene expression. Decreased *XPC* mRNA expression has been identified in human specimens from lung adenocarcinoma and lung squamous cell carcinoma, the two most common NSCLC histologic subtypes (119–121). Pre-clinical studies support epigenetic regulation of *XPC* with different environmental exposures, possibly due to promoter hypermethylation or histone-related transcriptional regulation (122). For instance, exposure of C57Bl/6 mice to 6 months of cigarette smoke led to decreased *Xpc* mRNA expression without altered expression of other studied BER and NER genes, including *Xpa* and *Ogg1* (66). XPC protein expression is decreased in lung fibroblast and bronchial epithelial cell lines treated in culture with cigarette smoke extract, but not other NER proteins including XPA, and may be due to protein turnover by ubiquitination (123). Tight control of XPC ubiquitination is likely required to ensure DNA repair but may be dysregulated in human cancers, including lung cancers, which have been shown to have high levels of ubiquitin ligases, such as Cullin-RING ubiquitin ligase 4 A (CUL4A), overexpression of which is common in cigarette smoke-related lung cancer, and which is inversely proportionate to *XPC* expression (26). Additionally, murine exposure to side-stream smoke (up to 4 months) and nicotine-containing e-cigarette vape (12 weeks) led to increased DNA adduct formation and decreased *Xpc* and *Ogg1* mRNA expression in the lungs (124, 125). Importantly, these studies also show decreased *in vitro* BER and NER repair using lysates from e-cigarette vape exposed mouse lungs, correlating decreased gene expression to decreased repair function.

The strongest evidence supporting a critical role of XPC in lung cancer comes from translational animal studies. Two mouse models of global *Xpc* deficiency have been created, both of which are associated with complete loss of functional XPC and cause characteristic skin cancers with exposure to UV light (126, 127). Increased DNA damage has been observed in the lungs of *Xpc* deficient mice upon exposure to oxidizing agents, but not in mice deficient in another NER protein, *Xpa*, although both show increased mutational frequency in the liver (40, 128). Exposure to urban air pollution led to increased lung inflammation and DNA damage in *Xpc* deficient mice (129). Mice homozygous deficient in *Xpc* develop lung tumors (primarily adenomas) with advanced age (16-17 months), although development of adenocarcinomas were rare without a co-existing loss of another tumor suppressor gene (130). However, exposure of *Xpc* deficient mice to chronic cigarette smoke and carcinogens, including urethane, MCA-BHT, 2-acetylaminofluorene (AAF) and NOH-AAF leads to lung adenocarcinoma development (42, 131), and with advanced age and chronic cigarette smoke, *Xpc* deficient mouse lungs develop an increase in lung compliance and alveolar rarefication similar to that seen in emphysema, a lung disease which predisposes to lung cancer (66). Importantly, mice heterozygous in *Xpc* (*Xpc+/-*) exposed to the carcinogen, urethane, developed an intermediate number of lung tumors when to compared to urethane-treated *Xpc* deficient and proficient littermate mice, suggesting a gene-dose effect and further supporting a role for intermediate levels of XPC expression, either through polymorphisms or epigenetic regulation, in lung cancer development (42).

Other more recently proposed mechanisms for XPC involvement in NSCLC development include regulation of cell proliferation and migration, and transcriptional regulation of p53. For instance, XPC, complexed with HR23B, impacts p53 transcriptional regulation of MMP1, low expression of which was associated with increased tumor size and metastasis (132). Cui and colleagues studied the impact of XPC on NSCLC cell lines *in vitro*, finding that *XPC* knock-down led to increased NSCLC cell growth and migration due to decreased surface e-cadherin expression through regulation of the SNAIL pathway (133). Although strong evidence supports an important role of XPC in lung cancer development, more research is needed to understand the link between alterations in XPC expression levels and XPC function on lung carcinogenesis and oncogenic development of characteristic genomic and transcriptomic alterations.

### Prostate Cancer

Prostate cancer (PC) is the most common malignancy in males (134), and *XPC* polymorphisms have been correlated to an increased risk of PC development in several studies (**Table 1**). For instance, the *XPC* polymorphism, *XPC* PAT (PAT I/I genotype) was associated with an increased odds of prostate cancer, associated with a 3.83-fold increased risk in a Tunisian population. In contrast, other *XPC* polymorphisms, including those heterozygous for Lys939Gln (939Lys/Gln) along with the PAT D/D haplotype are considered protective of prostate cancer (89). One more study reported an increased risk of developing PC in those with the *XPC* PAT polymorphism (PAT +/+ or PAT +/-) along with tobacco smoking in a Chinese population (90). Other studies have shown varied increases in PC risk with other *XPC* polymorphisms (91, 135) (**Table 1**). It does not appear that *XPC* polymorphisms are associated with more advanced disease in PC, and similarly, studies did not find an association between *XPC* gene polymorphisms and Gleason score (a measure of histologic PC staging which correlates to prognosis) (89, 92). However, using TCGA data, low *XPC* expression was associated with worse overall survival in PC, similar to analyses in many other solid organ tumors (135). These studies suggest that *XPC* polymorphisms may serve as a tool to identify those at the highest risk for developing PC, which can help in targeting high and low-risk individuals to appropriate screening and clinical evaluations.

### Ovarian Cancer

Like other solid organ tumors, *XPC* polymorphisms have been identified as one factor that may increase or decrease the risk of ovarian cancer as summarized in **Table 1**. Along with SNPs in two other NER proteins, XRCC1 and XRCC2, the *XPC* Ala499Val polymorphism was found to correlate to a decreased odds of ovarian cancer (OR 0.35) while the *XPC* Lys939Gln polymorphism was associated with an increased risk of ovarian cancer (OR 1.72) in a dominant genetic model (93). *XPC* polymorphisms may also serve as a biomarker in response to platinum-based chemotherapies as some specific SNP polymorphisms were associated with prolonged progression-free survival (PFS) (94). Going further, in ovarian cancer, overexpression of the eukaryotic translation initiation factor 3a (eIF3a) was associated with decreased response to cisplatin through downregulating *XPC* mRNA expression (136). This further supports an important role of XPC in predicting response to platinum-based chemotherapy through its canonical involvement in GG-NER.

### Bladder Cancer

As with several other cancers, DNA damage due to carcinogen exposure, including cigarette smoking, is strongly associated with bladder cancer. In this, as in several other cancers, *XPC* polymorphisms were associated with low penetrance susceptibility to bladder cancer (**Table 1**) (83, 86, 95–97). Several rare *XPC* mutations, identified in patients with bladder cancer, were studied *in vitro* and were associated with decreased XPC mRNA and protein expression (98). Supporting their likely role in bladder cancer development, XPC mRNA and protein expression is decreased in bladder cancer tumors and may portend a worse prognosis (99, 137, 138). A variable impact of factors such as cigarette smoking have been correlated to XPC expression in bladder cancers, and more recently, studies have suggested a role of both promoter hypermethylation and histone deacetylation by HDACs in regulation of *XPC* mRNA expression in bladder cancer (138, 139), the latter of which is supported by previously studies reporting SIRT-1 deacetylase regulation of XPC expression in other (skin) cancers (140). Overall, these studies support a role of XPC expression in variable risk and outcomes of bladder cancer, although the exact mechanisms of epigenetic regulation, and the specific mechanisms by which risk is altered in low XPC, remains less clear.

### Pancreatic Cancer

XPC may play a role as a risk factor for developing pancreatic cancer. As summarized in **Table 1**, some *XPC* polymorphisms have been described as increasing pancreatic cancer risk, particularly in smokers with the rs2470353 and rs2607775 variants (101). However, one study suggested a protective role of the *XPC*-PAT polymorphism (PAT +/+) in pancreatic cancer risk (100). Other studies suggested a role for genetic variants of other NER associated proteins, including ERCC1, but not necessarily XPC as a risk factor for developing pancreatic cancer (141). None-the-less, the specific role of NER, and specifically of XPC expression and epigenetic regulation, still need to be further explored in pancreatic cancer development.

### Other Solid Organ Cancers

In esophageal cancer XPC may play a role as a risk factor for developing malignancy. *XPC* genetic variants, specifically the *XPC* K939Q C/C genotypes were found to be associated with a higher mortality after treatment compared with patients with a wild-type homozygous genotype; particularly in those who were post-treatment with esophagectomy or neoadjuvant chemoradiation (102). Another polymorphism, *XPC* PAT +/+, was associated with decreased risk for esophageal cancer (103). The prognostic value of XPC is further supported by having two *XPC* polymorphisms, *XPC* 499CC and *XPC* 939AC+CC (939 Lys and Gln), as part of a 5-polymorphism panel (high risk genotype) that has a 79% sensitivity and 85.4% specificity of predicting 5 years progression free survival (104), indicating a potential prognostic role of *XPC* polymorphisms in esophageal cancer risk.

XPC may also play a role as a risk factor for other cancers including advanced colorectal cancer. The *XPC* polymorphism Ala499Val was found to play a protective role in developing advanced colorectal adenomas in smokers (105), and others have suggested a protective role of higher *XPC* mRNA and protein expression levels on colorectal survival, possibly related to an improved response to chemoradiation (106). A recent case-control association study using tissue from 493 breast cancer and 387 control cases suggested an association between two *XPC* polymorphisms, rs2228001-A>C (Lys939Gln) and rs2733532-C>T, with an increased odds of breast cancer (107), and another study with 200 cases and controls suggested an association between the *XPC* PAT+ allele and higher odds of breast cancer (108).

Finally, some evidence supports a role of XPC in liver (hepatocellular) carcinoma development. In a case-control study of hepatocellular carcinoma HCC related to aflatoxin B1 exposure, *XPC* polymorphism codon 939Gln allele, whether heterozygous (*XPC*-LG) or homozygous (*XPC*-GG), is associated with increased risk of HCC; these genotype variants correlated with decreased XPC tumor protein expression by IHC as well as a shorter overall survival (109).

## XPC as Tumor Suppressor and an Emerging Biomarker of Cancer Development

Numerous cancers are associated with decreased *XPC* expression, but the mechanism by which this occurs is less clear. The *XPC* gene, along with several other tumor suppressor genes, is located on chromosome 3p, a frequently site of chromosomal deletion in human tumors (130, 142). However, various modes of transcriptional regulation have been implicated in altered tumor *XPC* expression as well, and *XPC* expression may be altered in cells outside of the tumor itself. While studies have suggested decreased *XPC* expression in NSCLC tumor cells compared to surrounding lung (119), in 21 patients with NSCLC in which blood, tumor and lung tissue were collected, *XPC* mRNA expression was found to strongly correlate between blood and NSCLC tumor tissue, supporting the potential use of a minimally invasive blood draw as a prognostic and therapeutic biomarker (143).

The impact of low *XPC* mRNA expression may extend beyond alterations in DNA damage response and repair. Interestingly, XPC deficiency may also cause a mutational hot spot in the tumor suppressor p53 when treated with UV light, mediated by non-dipyrimidine base damage (144). Furthermore, there is evidence that XPC regulates a p53 post-ubiquitylation event and that XPC deficiency compromises p53 degradation, which may play a role in developing malignancy (145). These later two studies were performed in skin fibroblast cells and *in vitro* cell culture models, and whether XPC is involved in p53 regulation and mutations in other malignancies has not been well studied. In addition to its role in a number of DNA repair pathways, XPC has been implicated in transcriptional regulation both in response and independent of DNA damage. In the setting of DNA damage, studies have supported E2F1 transcriptional regulation of XPC expression (146). Recently, XPC itself has been implicated in post-translational histone modification and recruitment of transcription factors such as E2F1 to gene promoter sites independent of its regulatory role in DNA repair (69). High expression of *miRNA-346*, commonly elevated in NSCLC and other cancers, was associated with lower *XPC* mRNA and protein expression, indicating another potential mechanism for XPC downregulation in human cancers (147).

## XPC as a Biomarker of Response to Therapy

In addition to *XPC* polymorphisms and expression levels as potential biomarkers associated with risk for many malignancies, XPC may predict disease progression. In patients with NSCLC, low tumor *XPC* mRNA expression is associated with advanced stage at diagnosis and an increased rate of cancer relapse after treatment in never-smokers (148). Similarly in colorectal cancer, increased *XPC* expression was associated with longer 5 year survival in treated patients compared to patients with low *XPC* expression (106). *XPC* polymorphisms have been described as predicting response to platinum-based chemotherapy. For instance, DNA samples from whole blood cells showed that *XPC* rs2229090 GC/CC genotypes were associated with longer progressive free survival compared to the AA and GG genotypes (149). These findings are consistent with translational and *in vitro* studies inversely linking *XPC* mRNA expression with response to cisplatin, particularly in lung adenocarcinoma where cisplatin chemotherapy treatment remains a mainstay in locally advanced disease (150). However, a link between *XPC* polymorphisms and response to cisplatin therapy has not been clearly shown, with a recent meta-analysis (88). High mutational burden has been associated with improved response to the immune checkpoint inhibitors. Typically, angiosarcomas have poor response to immunotherapy, but a recent report highlights an angiosarcomas that developed in an XP-C patient which had the features suggestive of a good response to immunotherapy and ultimately benefitted from a good response to the immune checkpoint inhibitor pembrolizumab (151). This report provides a preliminary but intriguing potential link between XPC, high tumor mutation burden and response to immunotherapies.

In the last few years, more attention has been paid to targeting DNA repair as a modality to augment cancer therapy. For instance, in a micro-RNA (miR) screen of prostate cancer, *miR-890*, which directly inhibited transcription of *XPC* along with other DNA repair proteins, led to increased sensitivity to ionizing radiation, although further mechanistic testing indicated that IR-sensitization by *miR-890* persisted in *XPC* knock-down cells, suggesting an indirect role of XPC in double-strand break repair and overlapping gene-functionality in IR-sensitization (152). However, most studies show a predictive role in response to chemotherapies, especially platinum-based agents, which cause DNA lesions that are primarily repaired by NER, requiring XPC for recognition (2). Since increased NER repair could mean increased resistance to platinum-based therapy, inhibiting XPC could be a viable option to overcome platinum resistance in cancer cells. For instance modulation of XPC by hyperthermia or by treatment with sodium arenite was found to suppress XPC-induced cisplatin toxicity and sensitize tumors to platinum based therapy in a mouse ovarian cancer xenograft model (153). However, others have found a seemingly contradictory impact of histone deacetylase (HDAC) inhibitors in bladder cancer, showing a correlation between HDAC inhibition, increased XPC expression and higher cisplatin-induced activation of the pro-apoptotic protein, caspase 3 (139). Additionally, it is unclear if described decreases in XPC expression are in cancer cells alone or found in other cells within the tumor microenvironment, such as fibroblasts, in which XPC inhibition could be expected to decrease the tumor promoting cytokine IL-6 (72). On the other hand, this inhibition may help to sensitize tumor cells to other therapies due to the involvement of XPC in other DNA repair pathways and in checkpoint activation. Future studies should explore XPC targeting by small molecular inhibitors to investigate these possibilities, especially given conflicting data regarding XPC expression levels and therapeutic response to chemotherapeutic agents.

## Conclusion

XPC is increasingly recognized as playing an important role in the development of non-dermatologic malignancies. Decreased *XPC* mRNA and protein expression has been described in a number of cancers, with gene polymorphisms, deletions, and transcriptional regulation all active areas of research in the regulation of XPC expression. Additionally, research supports a role of XPC in the prognosis and treatment response in several of these cancers. Although XPC’s essential role in the recognition of bulky DNA lesions and subsequent activation of GG-NER, when altered, is a leading mechanism for development of UV-induced dermatologic malignancies and in modifications of cancer response to chemotherapies including cisplatin, recent data support a non-canonical role of XPC in DNA damage response and repair mechanisms, tumor suppressor transcriptional regulation, and in the development of non-dermatologic malignancies. Future studies would benefit from studying XPC as a biomarker of cancer prognosis and response to treatment in non-dermatologic malignancies.

## Author Contributions

Conceptualization, NN and CRS. Writing—original draft preparation, NN, BMW and CRS. Writing—review and editing, NN and CRS. Funding acquisition, CRS. All authors have read and agreed to the published version of the manuscript.

## Funding

This work was funded by the U.S. Department of Veterans Affairs BLR&D, Merit Review grant I01-BX005353 to CRS and in part by the National Institutes of Health, U.S.A. T32HL091816 to BMW.

## Author Disclaimer

CRS is employed by the U.S. Department of Veterans Affairs. The contents of this manuscript do not represent the views of the U.S. Department of Veterans Affairs or the United States Government.

## Conflict of Interest

CRS has served on Scientific and Medical Advisory Boards for Biodesix, Inc., bioAffinity Technologies, and as a scientific medical consultant for Bristol-Myers Squibb Company; these are not relevant to the topic of this manuscript. 

The remaining authors declare that the research was conducted in the absence of any commercial or financial relationships that could be construed as a potential conflict of interest.

## Publisher’s Note

All claims expressed in this article are solely those of the authors and do not necessarily represent those of their affiliated organizations, or those of the publisher, the editors and the reviewers. Any product that may be evaluated in this article, or claim that may be made by its manufacturer, is not guaranteed or endorsed by the publisher.
